# The Problem of Malnutrition Associated with Major Depressive Disorder from a Sex-Gender Perspective

**DOI:** 10.3390/nu14051107

**Published:** 2022-03-06

**Authors:** Cielo García-Montero, Miguel A. Ortega, Miguel Angel Alvarez-Mon, Oscar Fraile-Martinez, Adoración Romero-Bazán, Guillermo Lahera, José Manuel Montes-Rodríguez, Rosa M. Molina-Ruiz, Fernando Mora, Roberto Rodriguez-Jimenez, Javier Quintero, Melchor Álvarez-Mon

**Affiliations:** 1 Department of Medicine and Medical Specialities, University of Alcala, 28801 Alcalá de Henares, Spain; maalvarezdemon@icloud.com (M.A.A.-M.); oscarfra.7@hotmail.com (O.F.-M.); arb00031@red.ujaen.es (A.R.-B.); guillermo.lahera@gmail.com (G.L.); j_m_montes@hotmail.com (J.M.M.-R.); mademons@gmail.com (M.Á.-M.); 2 Ramón y Cajal Institute of Sanitary Research (IRYCIS), 28034 Madrid, Spain; 3 Department of Psychiatry and Mental Health, Hospital Universitario Infanta Leonor, 28031 Madrid, Spain; fernando.mora@salud.madrid.org (F.M.); fjquinterog@yahoo.es (J.Q.); 4 Psychiatry Service, Center for Biomedical Research in the Mental Health Network, University Hospital Príncipe de Asturias (CIBERSAM), 28806 Alcalá de Henares, Spain; 5 Psychiatry Service, Center for Biomedical Research in the Mental Health Network, University Hospital Ramon y Cajal (CIBERSAM), 28034 Madrid, Spain; 6 Department of Psychiatry and Mental Health, San Carlos Clinical University Hospital, IdiSSC, 28034 Madrid, Spain; rosamolina18@hotmail.com; 7 Department of Legal Medicine and Psychiatry, Complutense University, 28040 Madrid, Spain; rodriguez.jimenez.psiquiatra@gmail.com; 8 Institute for Health Research 12 de Octubre Hospital, (Imas 12)/CIBERSAM (Biomedical Research Networking Centre in Mental Health), 28041 Madrid, Spain; 9 Immune System Diseases-Rheumatology, Oncology Service an Internal Medicine, University Hospital Príncipe de Asturias, (CIBEREHD), 28806 Alcalá de Henares, Spain

**Keywords:** depression, stress, malnutrition, deficiencies, sex differences, menstrual cycle, premenstrual syndrome, premenstrual dysphoric disorder, pregnancy, postpartum, menopause

## Abstract

Major depressive disorder (MDD) is an incapacitating condition characterized by loss of interest, anhedonia and low mood, which affects almost 4% of people worldwide. With rising prevalence, it is considered a public health issue that affects economic productivity and heavily increases health costs alone or as a comorbidity for other pandemic non-communicable diseases (such as obesity, cardiovascular disease, diabetes, inflammatory bowel diseases, etc.). What is even more noteworthy is the double number of women suffering from MDD compared to men. In fact, this sex-related ratio has been contemplated since men and women have different sexual hormone oscillations, where women meet significant changes depending on the age range and moment of life (menstruation, premenstruation, pregnancy, postpartum, menopause…), which seem to be associated with susceptibility to depressive symptoms. For instance, a decreased estrogen level promotes decreased activation of serotonin transporters. Nevertheless, sexual hormones are not the only triggers that alter neurotransmission of monoamines and other neuropeptides. Actually, different dietary habits and/or nutritional requirements for specific moments of life severely affect MDD pathophysiology in women. In this context, the present review aims to descriptively collect information regarding the role of malnutrition in MDD onset and course, focusing on female patient and especially macro- and micronutrient deficiencies (amino acids, ω3 polyunsaturated fatty acids (ω3 PUFAs), folate, vitamin B12, vitamin D, minerals…), besides providing evidence for future nutritional intervention programs with a sex-gender perspective that hopefully improves mental health and quality of life in women.

## 1. Introduction

Major depressive disorder (MDD), commonly known as depression, is an incapacitating global health problem characterized by altered mood, including loss of interest and pleasure, as well as impaired cognitive and vegetative functions [1]. This disorder affects at least 3.8% of the population and it is estimated that there are approximately 290 million new cases per year [2], becoming one of the six drivers that increased the global burden of disease in the period from 1990 to 2019 [3]. Likewise, according to the projections foreseen for 2030 by the World Health Organization (WHO), MDD will lead the group of disabling diseases [4].

In addition, depression leads to a prolonged disturbance in daily activities [5] that, consequently, will be associated with both direct and indirect costs. Presenteeism and absenteeism accounted for around 50% of the USD 210 billion of total spending in the US in 2010. Although the indirect costs imply a greater economic impact, we must consider the direct costs, which involved an outlay of USD 98.9 billion in the same year [6]. Likewise, according to the report carried out by the WHO in 2007 the socioeconomic costs in Europe follow the same trend, accumulating an amount of EUR 136.3 billion invested, with around 70% of this attributable to the lack of productivity [7]. 

A greater number of women with depression have been detected compared to men; the ratio remains at 2:1 [8]. The main source of this difference has been found in the biological factor; it is estimated that the heritability of this disorder ranges between 30 and 40%, assuming a greater probability of inheritance in women compared to men [9,10]. Moreover, the patient’s vulnerability to external factors during the course of depressive episodes will be increased by the action of sexual hormones and the repercussion of the oscillations will be significant and will change depending on the age range [11]. Thus, for example, in adolescence, the initial moment of activation of these hormones and the start of menstruation are a focus in the case of girls, who suffer higher levels of chronic stress than boys [12]. Fluctuations in ovarian hormones could be one of the potential causes of the disparity in prevalence between the sexes, since the group in question develops forms of depression such as premenstrual syndrome (PMS), postpartum depression and postmenopausal depression [13]. Certain cycle imbalances such as heavy bleeding have been significantly associated with the diagnosis of MDD [14]. In addition, MDDs trigger the appearance of suicidal thoughts in at least 60% of adolescents [15], making it the second leading cause of death in this group [16]. These events have also been related to the first menses of puberty in girls [17].

On the other hand, the impact of socioeconomic aspects, age, race and the presence of other comorbidities cannot be ignored [18]. In this sense, it has also been shown that the risk of suffering from MDD worsens when the framework of difference in political participation or economic independence is less egalitarian, leading to a clear increase in symptoms, as well as persistent depression [19,20]; if we find ourselves again in the range between 14 and 25 years, the dissimilarity will be more accentuated between both sexes, while it is not so evident in older age groups [21].

Going deeper into the investigation of MDD in women would help to decipher the causes of the events, thus providing new management and treatment tools. It is true that the patient’s nutritional status is closely related to depression and may be a key tool for its prevention and treatment [22]. In this line, repeated eating patterns and specific physiological situations have been observed in the case of women (menstrual cycle, childbirth, postpartum, menopause), which lead to marked nutritional deficiencies, especially micronutrients such as vitamin D, omega-3 fatty acids (FA ω-3), methyl-folate and S-adenosyl methionine (SAMe). These components have also been the object of study in preclinical and clinical trials, standing out for their benefit as potential adjuvant nutraceuticals in patients with MDD [23,24].

The main objective of this paper is to critically and descriptively review the impact of malnutrition on the pathophysiology of MDD in women, also evaluating possible strategies based on nutritional intervention that may be helpful in limiting the progression or promoting recovery from depression, as well as preventing the appearance of future depressive episodes.

## 2. Major Depressive Disorder in Women

### 2.1. General Pathophysiology 

#### 2.1.1. Can MDD Be Located in the Brain?

One of the big questions that many researchers have historically asked is: Can MDD be located in the brain? Unlike other neuropsychiatric diseases that affect specific regions or mechanisms of the nervous system, in MDD, the alteration is not located in any specific region. In contrast to other diseases where the main organ affected is the brain, in MDD the alteration is not localized, thus affecting different parts of this organ.

Several investigations show structural and functional alterations; the findings are mainly presented in the fluctuations in the amount of gray matter in the frontal and parietal lobes, hippocampus, amygdala, corpus striatum, caudate nucleus, thalamus and cingulum [25,26,27]. Wei Peng et al. determined in their study that gray matter volume abnormalities in the fronto-limbic region of the brain are basic evidence of depression in the first episodes of patients without prior medication. Gray matter mass in this situation has been found to increase in the middle superior frontal gyrus, cuneus, left paracentral lobe and bilateral thalamus; however, it decreases in the region of the left insula, middle frontal gyrus, superior frontal gyrus and right dorsolateral [28]. The frontal lobe is considered to be the area that undergoes the most changes in patients with MDD. The thickness of the anterior cingulate cortex (ACC) is modified. It has been shown that in patients with treatment-resistant depression, the thickness increases when depressive episodes subside over time, with the opposite effect occurring in recurrent patients [29]. 

On the other hand, the thalamus is a structure formed by different nuclei connected to the cerebral cortex, as well as the amygdala, hippocampus and striatum. It is closely linked with the regulation of emotions, memory control and arousal [30]. Likewise, it intervenes directly in the maintenance of circadian rhythms and the generation of sleep-wake patterns [31]. In patients with depression, it has been shown that there is a decrease in volume due to loss of gray matter [32] and changes in its shape. This last parameter maintains a direct relationship with the classification of the severity of depression proposed by Hamilton (HAM-D) [33]. 

In addition, it should be noted that the volume of the hippocampus is reduced by up to 1.2% in people with recurrent depressive episodes or when the onset of the pathology manifests itself at an early age, not showing this difference in the first episodes or in people with late onset [34]. Atrophy occurs as a consequence of an increase in glucocorticoid levels due to the alteration of the hypothalamic-pituitary-adrenal (HPA) axis [35]. Moreover, it has been proven that functional and structural changes in this brain region lead to abnormal emotional processing and defective cognition [36].

#### 2.1.2. Cellular and Molecular Changes Associated with MDD

The alteration of the reorganization capacity of the nervous system induced by the response to stimuli can be explained with the neurotrophic hypothesis of MDD, which postulates that the reduced expression of growth factors triggers neuronal atrophy, leading to decreased neuroplasticity, as well as errors in the neurogenesis of brain regions responsible for regulating mood and memory [37]. Neurotrophic factors intervene in the regulation of the plasticity and formation of neuronal networks [38]. The brain-derived neurotrophic factor (BDNF) belongs to this family and is involved in the modulation of neurogenesis, synaptic plasticity, as well as basic neuronal functions [39]. It is possible that this factor triggers both depressive and antidepressant behaviors, obeying the BDNF signaling regulation network, although it is true, even though it is a key element, that the isolated interruption of its signaling is not enough to result in the spectrum of depressive symptomatology [40]. There is a close relationship between stress and changes in the expression and signaling of BDNF in different brain regions (amygdala, hippocampus, prefrontal cortex, etc.); these alterations are correlated with the appearance of depressive behavior [41]. 

In the same context, cortisol (steroidal hormone or glucocorticoid) plays a substantial role in the development of the pathophysiology of depressive disorder. Under the effect of a stressful stimulus, the HPA axis will be activated by the secretion of corticotropin-releasing factor (CRF), arginine-vasopressin (AVP) and other neuropeptides. As a consequence, the adrenocorticotropic hormone (ACTH) is produced, which, in turn, stimulates the adrenal glands, synthesizing them as cortisol. In patients with MDD, hypercortisolemia leads to overstimulation of the HPA axis, followed by a clear disruption of neurotransmitter metabolism [42].

In this sense, it is of great importance to know the monoaminergic hypothesis, which maintains that there is a decrease in monoamines in the synaptic space due to disturbances in physiological mechanisms; that is, low sensitivity of the receptors, alteration of the secretion to the synaptic space or storage failure [43]. Until now, the *monoamine hypothesis* has been a pioneer as the basis for the target of the development of antidepressants. One of the keys to the pathophysiology of this condition lies in the serotonin transporter membrane protein (SERT), as well as neuronal serotonin receptors [44]. It has also been shown that norepinephrine and its alpha-adrenergic receptors are highly relevant in the regulation of cognitive development or response systems to adverse situations, such as fear or anxiety [45]. Similarly, understanding the disruption of the dopaminergic system mechanisms is the key to mediating characteristic symptoms of this disease, such as anhedonia [46]. On the other hand, low concentrations of other types of neurotransmitters, such as glutamate, GABA or acetylcholine, have been shown in patients with MDD compared to controls, which opens up an interesting research opportunity for more effective therapies [47]. Oxytocin (OXT) and AVP, whose release is promoted by anxiogenic, stressful and notably social stimuli, both positive and negative, have also become attractive research targets [48,49]. The predominance of one of these neuropeptides has a direct impact on the other and vice versa; this could be explained by the benefits obtained by inhibiting AVP so that the balance shifts towards OXT secretion in order to improve depressive behavior [50].

#### 2.1.3. Systemic Alterations Associated with MDD

As can be seen, major depressive disorder has a large network of interconnected mechanisms that give rise to its pathophysiology, which is why other fields such as the immune system have been investigated more thoroughly. A wide variety of proinflammatory cytokines, such as IL-1β, IL-2, IL-6, IL-12 and tumor necrosis factor (TNF-α), in addition to some antibodies (ribosomal antibody-P and anti-N-receptor methyl-D-aspartate) interfere in the mediation of the inflammatory processes of depression [51]. The lesions produced in the central nervous system, mainly due to stressors, will trigger the activation of microglia and this will lead to the start of the immune cascade. During this process, there will be an increase in the secretion of cytokines, which could pass the blood-brain barrier and, as a consequence, increase blood levels, thus becoming highly relevant markers. In addition, this dysregulation will contribute to an exacerbated activation of the HPA axis, giving rise to the cycle of pathological mechanisms characteristic of depressive disorder [52]. 

In this sense, there is evidence that suggests the close relationship between the immune system and the intestinal microbiota, an example of which is the production of immunocytokines in response to the metabolism of intestinal bacteria [53]. The overstimulation of the HPA axis will lead to an increase in the permeability of the intestinal barrier; subsequently, a phenomenon known as endotoxemia occurs, in which the bacterial endotoxins pass into the blood circulation, triggering the onset of peripheral inflammation; the signals will arrive at the CNS via the vagus nerve, again promoting the neuroinflammatory response of glial cells [54]. This fact will lead to a greater systemic deterioration of the subjects with depression [55].

**Figure 1:**
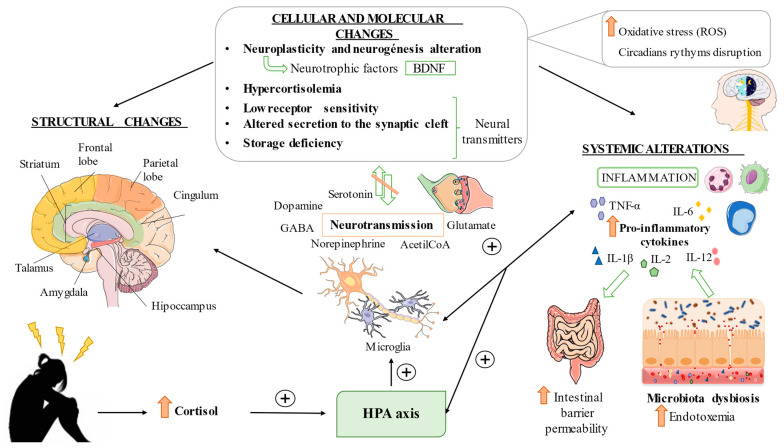
General pathophysiology of MDD. Elevated stress affects the HPA axis involving cellular and molecular changes, resulting in brain structural and functional changes. For instance, abnormal neurotransmission, microglial activation, neuronal damage and dysregulation of neuroplastic and neurotrophic factors can be reported in patients with MDD. These alterations are frequently accompanied by enhanced oxidative stress and circadian rhythms disruption. Exacerbated systemic inflammation and gut dysbiosis and enhanced intestinal barrier permeability are also major characteristics of patients with MDD. It is of note that all these mechanisms boost each other, perpetuating the damage and pathologic environment related to MDD.

Other factors also intervene in the pathophysiology of MDD; since time immemorial, it has been suspected that sleep is essential to good health. This idea led, decades ago, to the search for keys to understanding depression, and a plethora of studies argue for the disruption of circadian rhythms in patients with MDD [56,57]. Additionally, the brain is especially vulnerable when we talk about the action of reactive oxygen species (ROS), as this is a structure with high content of oxidizable lipids and an oxygen requirement. It has been observed that there is an increase in ROS as a consequence of the alteration of the antioxidant power of cells in depressive subjects. Due to this, it has become another of the factors in the investigation of this pathology [58,59]. A summary related to the three main points herein exposed can be found in Figure 1.

### 2.2. Pathophysiology Specific to MDD in Women

#### 2.2.1. Biological Mechanisms

It has been observed that there is divergence in molecular processes in both sexes. In the case of women, there is an increase in genes linked to neuronal synapses, as well as a reduction in markers of immune function and microglia, in contrast to the findings observed in men [60]. In accordance with the above, different investigations agree that the pro- and anti-inflammatory response is attenuated in women with depression and there is greater reduction in those with more severe symptoms [61,62]. A possible explanation for this finding is an increase in tolerance due to pre-exposure to endotoxins due to the imbalance of the intestinal barrier [61]. Moreover, the recent study by Chen et al. has observed significant differences in the microbiota according to the sex of the patient; among other things, it is possible to relate the severity of the pathology to the state of the microbiota, finding a negative relationship in women when there is a predominance of *Streptococcus* or *Clostridium* [63]. The latter plays an important role in the regulation of non-ovarian estrogen levels, affecting the risk of suffering from conditions of a different nature in postmenopausal women and elderly men [64].

In this sense, and based on new findings, it is worth highlighting the role of the estrobolome, which is the set of genes belonging to intestinal microorganisms responsible for modulating estrogen levels in the blood through various mechanisms [65]. The appearance of contemporary depressive episodes with hormonal fluctuations is evident, which are manifested above all in puberty, postpartum or menopausal transition periods [66,67,68]. In the follicular phase and in perimenopause, where normal levels of estradiol (E2) are low, a greater vulnerability when receiving stressful events or negative emotions has been observed, all due to hormonal disturbance of the activity of the structures involved in affective regulation; the opposite occurs in phases of the cycle with high levels [69]. Several pieces of evidence confirm the relationship between hormones and the estrobolome; for example, genera that produce short-chain fatty acids, such as *Prevotella*, *Ruminoccocus* and *Roseburia*, decrease in postmenopausal women compared to premenopausal women [70]. Moreover, animal models reflect the existence of a change in the intestinal microbial composition due to the hormonal changes typical of menopause [71]. In the same way, the induction of progesterone in ovariectomized mice led to an improvement in depressive behavior, all of which was related to the increase in Lactobacillus and the decrease in IL-6 [72].

On the other hand, we will discuss the pathophysiology of specific conditions that occur during these fluctuations in female gonadal steroid hormones. PMS and premenstrual dysphoric disorder (PMDD) occur during the luteal phase of the menstrual cycle and are related to progesterone, which, in turn, could interact with GABA and 5-HT by changing the configuration of their receptors; as a consequence, there will be a pause of its activation [73]. However, there are more mechanisms involved, but the path of the disorder is not known with certainty [74]. Another variant of MDD, postpartum depression (PPD), has hormonal, genetic and immunological precursors implicated in its pathophysiology. A clear biomarker is allopregnanolone, a progesterone metabolite with anxiolytic and antidepressant power, whose low levels after childbirth have been associated with an increased risk of developing PPD. One possible explanation is its interaction with GABA receptors [75]. Likewise, in premenopausal depressive disorder, progesterone levels remain constant [76] but E2 stands out when its levels are higher, assuming a direct risk of the intensification of depressive symptoms at this stage [77].

**Figure 2:**
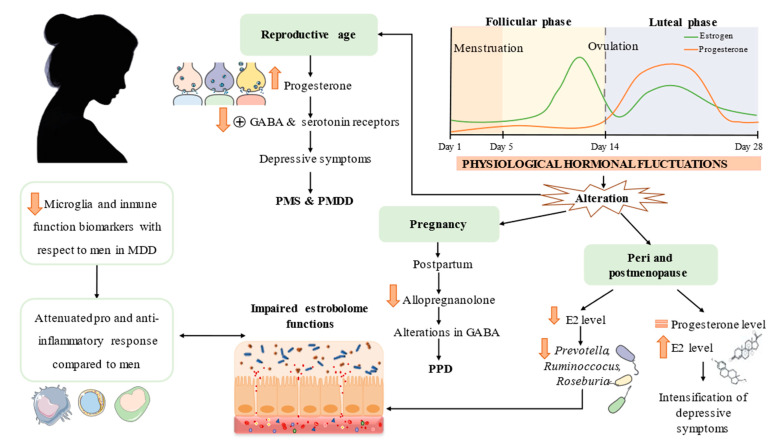
Hallmarks in women-specific MDD pathophysiology. Different moments in a woman’s life entail different hormone fluctuations with consequences at the neurotransmitter level. These changes also affect estrobolome functions and positively correlate with MDD severity. Different immune response compared to men is also emphasized. MDD = major depressive disorder; PMS = premenstrual syndrome; PMDD = premenstrual dysphoric disorder; PPD = postpartum depression; E2 = estradiol.

Biological particularities related to women in different moments of life are briefly shown in Figure 2.

#### 2.2.2. Psychological and Sociocultural Factors

It must not be forgotten that there are plenty of non-biological factors that help to explain the different rates of MDD in women in comparison to men. As mentioned above, psychological stress is a major driver of depression, associated with multiple structural and functional changes in the brain. Chronic stress and early life stress (ELS) are frequently involved in the onset and development of depression independently from sex-gender [78,79]. However, different studies have observed that there is a differential response in the brain between men and women, and also the perceived origin stress can be different in both populations. ELS comprises various forms of child abuse and neglect affecting a large number of children worldwide, being closely related with the development of several psychiatric disorders [80]. Prior mice models have suggested that women appear to be more sensitive to the detrimental effects of ELS, showing an early emerging female-specific depressive phenotype and a rapid response to antidepressants [81]. For instance, it is known that despite the hippocampus not being affected due to ELS in females, the reward system may be altered, explaining the differential long-term consequences of ELS observed in women, including the higher risk of MDD [82,83]. On the other hand, women with higher levels of chronic stress are more prone to suffer from depressive episodes, especially after an occurrence of acute stress [84]. Interpersonal relationships can be considered a major source of stress and depression risk in women, as depressed women often inhabit a complicated interpersonal environment, including divorce, marital problems or difficulties with their children [85,86]. Other psychosocial factors implicated in the onset and development of MDD in women include negative and ruminative thinking, low self-esteem, depression stigma and the diagnosis of a chronic disease, with an inverse relationship with spiritual well-being [87].

Last but not least, there is no denying the fact that cultural dictation of female roles is one of the key determinants of depression in women, along with the lack of emotional and social support [88]. The influence of some traditions or religions and immigration in the higher prevalence of depression in certain groups of women is of note [89,90,91]. A low socioeconomic status, especially at old age, is directly correlated with a greater risk of depression in women [92]. Educational level, occupational situation, sexual satisfaction and even the neighborhood or living in rural versus urban areas can also influence the development of depression in women [93,94,95,96]. Despite depression affecting virtually all ethnic groups, there are some disparities in the prevalence and treatment-seeking behavior across race [97]. 

Overall, there are numerous psychological, social and cultural factors associated with the development of depression in women. Besides, these factors appear to be frequently accompanied and exert a cumulative effect on depression (i.e., ELS causes functional and structural changes in the brain that may be related with interpersonal difficulties, low self-esteem, professional difficulties and other factors with detrimental effects on the life of women). All these multiple factors involved in the onset and pathophysiology should be considered collectively in the clinical management of women with MDD in order to properly address the different causes and consequences of depression. 

### 2.3. MDD Clinic

#### 2.3.1. General MDD Manifestations and Approaches

The diagnostic criteria proposed by the “*Diagnostic and Statistical Manual of Mental Disorder* (DSM-V)” state that there must be a deficit in the functional capacity of the individual that lasts for more than two weeks, a period during which there is a continuous manifestation of five or more symptoms, among which we must include the loss of reactivity to pleasant stimuli, which is usually caused by the daily activities carried out, and the presence of a sad or irritable mood, which we find in some cases of children and adolescents. These main criteria are accompanied by secondary disturbances, both somatic, including insomnia and hypersomnia, loss of energy, changes in appetite or weight, and non-somatic, for example, poor concentration or the appearance of suicidal thoughts [98]. The clinical guidelines for the diagnosis should focus on aspects related to the repetitiveness of the episode (one-off or periodic), improvement of symptoms, attention to the appearance of psychotic symptoms, as well as their severity^6^. To make a quantitative evaluation of this last parameter, the Hamilton Depression Rating Scale (HAM-D) is used, which stipulates a question for each clinical criterion that will be given a certain score, the sum of which will be between the values of 0 and 56 [99]. According to Zimmerman et al. various reference ranges have been established; a score of 0–7 is considered a non-depressive state; 8–16 is diagnosed as mild or moderate depression; at 17–23, the patient suffers from severe MDD, while values > 24 are categorized as very severe depression [100]. 

To address the development of the disease until its remission, non-pharmacological treatments are generally used, such as psychotherapy, as well as pharmacological ones. Individualized management is attempted, although it is classified according to the stage in which the patient is. In states of mild depression, no facts have been found that affirm that the use of drugs is beneficial, so active monitoring of the patient is recommended, as well as moderate psychological intervention [101]. Several studies show that in the case of moderate MDD, the use of psychotherapy gives better results than the isolated use of drugs, although it is true that the combination of both methods is the most effective on this occasion [102]. Likewise, in severe states of depression, the use of psychological techniques, such as cognitive behavioral therapies, together with the prescription of antidepressants, is strictly recommended [101]. In approximately 30% of individuals the first two antidepressant trials do not work adequately, which is known as treatment-resistant depression. In these cases, complementary therapies should be applied, such as the use of second-degree antipsychotics (SGA) or electroconvulsive therapy with remission effectiveness of 75% of cases [103]. In the same way, recent lines of research have put forth a potential proposal for hormonal therapies as a complementary treatment [104]. 

#### 2.3.2. Women’s MDD Clinic

MDD has twice the incidence in the female population compared to the male [2]. This fact is also reflected in the fact that the prescription of antidepressants is around 17.2% in women, while 8.2% of male patients receive this treatment [105]. As previously mentioned, ovarian hormone fluctuations could be one of the potential causes of the disparity in prevalence, giving rise to unique clinical presentations such as PMS, postpartum depression (PPD) and postmenopausal depression (PMD) [13].

Studies from the end of the last century have already revealed the differences that major depression presents in relation to the sex of the patients [106,107]. There is clear evidence of the symptomatologic disparity; in women a greater presence of some classic symptoms of this disorder has been observed, such as loss of interest or alteration of the sleep cycle, added stress and irritability, both associated with a bad mood. Increased appetite, linked to weight gain, or the appearance of obsessive thoughts is very representative in this group [108]. In addition, in women, the appearance of anxiety disorders is frequent in MDD, while in men it has comorbidity with substance use. The severity of the symptoms is greater in the female population; however, the appearance of more episodes in the other group stands out [109]. In the same way, it was deduced that the prescription of drugs that stimulate immune function would be convenient for female patients, while antidepressants that reduce immune activation are indicated for males [60]. It cannot be neglected that the factors that confirm the differences between the responses to treatment are not decisive at all, although it is stated that the efficacy of serotonergic antidepressants is increased in women compared to men, and another important observation is that women in the postmenopausal period experience a decrease in response compared to those of a younger age [110].

## 3. The Importance of Malnutrition in MDD from a Sex-Gender Perspective

Malnutrition in general encompasses various manifestations that reflect a poor diet, both in terms of lack and excess, leading to an imbalance between energy intake and expenditure [111]. In this sense, the relationship between depression and poor nutritional intake has been investigated, confirming the existence of this; however, in all the studies, they highlight the need to continue investigating this aspect further, due to the limitations of the studies carried out [112,113,114]. 

Eating behavior in women seems to play an important role. A recent study suggested that 13% of participants with MDD had lifelong eating disorders and 39% showed clinically significant disordered eating behavior, versus 3% and 11%, respectively, of participants without diagnosed depressive disorders. Therefore, the authors concluded that the rate of altered eating behaviors is higher in women with depression [115]. Along these lines, it is well known that emotional hunger is one of the basic characteristics of eating disorders [116]. The oscillations of ovarian hormones could have a close link with “emotional hunger” and the desire for more palatable foods rich in sugars. It has been observed in girls that high levels of leptin in the blood are related to high stress [117]. Several investigations in women have investigated the existence of differential responses to appetizing food throughout the cycle, concluding that in phases with a high concentration of estrogen, motivation for reward is lower, so emotional hunger is reduced; on the contrary, in the luteal phase where the predominant levels are those of progesterone, the desire for appetizing food increases exponentially [118]. Women more susceptible to binge eating have compromised interoceptive awareness of physiological states [119], resulting in dietary limitation that, as a consequence, could negatively affect the dopaminergic system [118]. 

However, binge eating is not the norm in MDD in general. On the other hand, we know that, especially in depressed older people, there is a great tendency to loss of appetite, skipping meals and involuntary weight loss [120,121], due to hypoactivation of the middle insular cortex. It would be interesting to carry out studies in which, when monitoring pregnant women, it can be detected if their frequent lack of appetite or other irregularities [122] are related to prevalence of postpartum depression; or to determine if, in women with postpartum depression, there is a tendency towards loss of appetite or binge eating in pregnancy.

From this association between eating disorders and MDD, the terms “depression-related increased appetite” and “depression-related loss of appetite” can be found in the scientific literature [123]. In any case, different eating patterns will lead to excesses in certain macronutrients and deficiencies in others, as well as deficiencies in certain micronutrients that seem to play important roles in the patient’s homeostasis.

### 3.1. Macronutrients

Macronutrients encompass carbohydrates, proteins and fats, and they play a central role in cognition and brain functioning [124]. In the case of women, macronutrients consumption appears to vary across the menstrual cycle. For instance, an increased intake of protein, particularly animal protein, can be reported during the luteal phase of the menstrual cycle [125], with more carbohydrate intake per day after ovulation [126]. These variations are due to sexual hormonal fluctuations and, in turn, adequate food intake is crucial for ensuring the functioning and actions of these hormones in tShe body [127]. In this part we will summarize the role of macronutrients and their deficiency in the onset and progression of MDD, PMS, PPD and PMD.

In recent decades, carbohydrate consumption has been stigmatized and closely linked to mood. The mechanisms that allow this interaction appears to be linked to the synthesis of different neurotransmitters, although special emphasis is placed on the serotonergic system due to the ability of insulin to increase tryptophan bioavailability in the brain [128]. There seems to be a clear derangement of glucose metabolism in patients with MDD [129], and high glycemic blood levels have been linked to suicidal behavior [130] and disruption in brain networks [131]. Despite the widespread belief that sugar consumption is necessary for the brain and improves mood, compelling evidence refutes this hypothesis, also suggesting projected harm in greater fatigue, a symptom associated with MDD [132]. Conversely, diets with poor carbohydrate intake could also be counterproductive, causing compromised metabolism [133]. There have been some studies conducted in women correlating carbohydrate consumption, mood and the risk of suffering PMS. Houghton et al. [134] found a direct association between maltose intake and the development of PMS, although high dietary glycemic index was found to be correlated with decreased premenstrual symptoms in a group of young Japanese women [135]. In the same line, high dietary glycemic index but not glycemic load was found to be associated with fewer depressive symptoms in young and middle-aged women [136]. It should be remarked here that the origin of the dietary carbohydrates should be deeply studied, although it seems that high dietary glycemic index may benefit women’s mood and brain function in reproductive ages. However, further studies are required, as there are other works suggesting the opposite conclusions without considering a sex-gender perspective [137,138]. On the other hand, the evidence has failed to find any relationship between carbohydrate intake and PPD [139,140]. Besides, a study carried out in a cohort of postmenopausal women revealed the negative influence of diets rich in refined carbohydrates, which consequently increase the risk of depression [141]. Conversely, high consumption of fiber appears to be directly correlated with sex-hormone-binding globulin (SHBG), a protein involved in sex hormone transportation. Interestingly, in postmenopausal women, SHBG and sex hormone levels have been associated with depressive symptoms, although more efforts are needed in this field [142].

Proteins also play a vital role in multiple biological processes. They are molecules formed by amino acids linked together forming chains; the products derived from the hydrolysis of these will be used by the bacteria of the digestive system, by the cells for the synthesis of metabolites involved in relevant physiological processes and are destined to play a role in the formation of other tissues such as the muscle [143]. Animal studies have helped us gain further insights into the negative effects of deficient protein intake. For instance, greater susceptibility has been observed in female mice in developing depressive symptoms at an early age when they have been deprived of protein intake in the perinatal stage [144]. Likewise, there was loss of neurons and alteration of the precursor genes of hippocampal neuroplasticity in young adult female mice with protein malnutrition [145]. In addition, not only the amount of protein but also its quality is essential for a healthy diet [143]. Despite some studies reporting a negative association between excessive protein intake and depressed mood in women [146], no relationship has been found between protein consumption and PMS [147]. More studies are needed to evaluate the effects of protein intake and depression in women.

Another of the macronutrients that are linked to depression is lipids. It seems that women are in general more prone to having intakes that exceed recommendations for total and saturated fat [148]. First of all, it should be highlighted that trans fatty acids, as poor-quality lipids in the induction of dysbiosis, promote the proliferation of harmful bacteria such as Proteobacteria and reduce those that provide benefits to metabolic processes [149]. Thus, in premenopausal women, a positive link between high trans fatty acids intake and depressive symptoms has been reported [150]. Likewise, their consumption seems to increase proinflammatory responses [151] and enhance the permeability of neuronal membranes, implying the compromise of the dopaminergic system and the appearance of anxious symptoms [152]. On the other hand, there is evidence of the benefits provided by some, such as omega 3 polyunsaturated fatty acids (ω-3 PUFAs), specifically eicosapentanoic acid (EPA) and docosahexaenoic acid (DHA). Mainly, two physiological mechanisms associate them with depression; EPA and DHA, being part of the cell membranes of neurons, are crucial for the correct neurotransmission and cell signaling and, in turn, have the power to inhibit the proinflammatory cytokines characteristic of patients with MDD and inflammatory eicosanoids that are elevated in these cases [153]. Women with MDD usually present low intake of ω-3 PUFAs, which is inversely correlated with depressive symptoms and is directly associated with brain functional connectivity [154]. Besides, the benefits from increasing ω-3 PUFAs have also been reported in PMS, PPD and PMD, although further studies are required to confirm these results [155,156,157]. Linked to the low intake of ω-3 PUFAs, the consumption of omega 6 fatty acids (ω-6 FAs) is increased in Westernized diets. Over the decades, the ratio has changed from a balance of both fatty acids to ω-6/ω-3 ratios between 10:1 and 20:1. These long-chain polyunsaturated lipids are derived from linoleic acid and share the enzymes (elongases and desaturases) that are involved in their biosynthesis. Consequently, abundant consumption of ω-6 PUFAs interferes with the desaturation of ω-3 PUFAs, thus leading to loss of homeostasis, promoting the genesis of cardiovascular, inflammatory or cancer disorders, among others [158]. High ω-6 PUFAs intake appears to be linked with a higher risk of depressive symptoms among pregnant women, with total fatty acids intake and omega-6/omega-3 ratio above recommendation [159] also being predictive as a potential risk factor of later PPD [160]. In this sense, the fatty acids in question stand out for their role in the regulation of inflammatory pathways. The association of the ω-6/ω-3 imbalance has been found with the increase in IL-6, exacerbating inflammatory reactivity; therefore, it will mean greater vulnerability to the appearance of depressive symptoms [161]. The reversal of the balance towards the fatty acids balance could be beneficial for the improvement of subjects with MDD.

### 3.2. Micronutrient Deficiencies Related to Reproductive Age, Postpartum and Later Life

Firstly, vitamins are a group of essential micronutrients that appears to be affected in women with MDD. Vitamin D (Vit D) is a fat-soluble hormone, known for its role in calcium homeostasis and bone growth. It also plays a decisive role in neurological development. The synthesis and elimination of this micronutrient, as well as vitamin D receptors (VDR), are found in the brain [162]. Consequences of vitamin deficiency that have been related to the pathogenesis of MDD are alterations in the hippocampus in animal models [163] and imbalance in neuronal excitation and inhibition pathways [164]. Evidence has been found of the improvement that this nutrient produces in depressive symptoms; however, it is not so conclusive that adequate concentrations can prevent the development of depression [165].

Among the processes in which vitamin B12 is involved, DNA synthesis, blood cell synthesis and neurological functions stand out for their relevant action [166]. Interdependence with folate increases the processes in which B12 interferes [167]. Research, both in childhood and adolescence as well as in adulthood [168], shows that these two micronutrients, as well as homocysteine, are involved in one-carbon metabolism and methylation processes of neurotransmitters, proteins and phospholipids of the cell membrane. Low concentrations of B12 and folate are linked to the alteration of the above [169]. Moreover, in this situation, the production of a neurotoxic metabolite of homocysteine is stimulated; as a consequence, there is overstimulation of NMDA receptors that increases the flow of intracellular calcium, increasing oxidative stress and apoptotic responses [170]. All this leads to the contribution of the pathogenesis of MDD [168].

On the other hand, women with MDD seldom meet mineral requirements. Several studies point to a usual low serum zinc level in patients with MDD [171]. The influence of the micronutrient on the glutamatergic system has been demonstrated, producing the inhibition of the N-methyl D-aspartate (NMDA) receptor via the zinc receptor GPR39, a process necessary to preserve glutamate and GABA homeostasis [172]. Likewise, it is stipulated that part of its benefit lies in its ability to activate neuronal plasticity processes and its interaction with monoamines [173]. Selenium (Se), among other things, is part of the selenoproteins highly involved in the signaling of redox processes [174]. In this line, other organic compounds containing selenium have been investigated, such as, for example, 3-((4-chlorophenyl) selanyl)-1-methyl-1H-indole (CMI), which has shown results regarding its inhibitory power on the inflammation associated with oxidative stress in in vitro studies [175] and in mice models [176]. The cross-sectional study by Li et al., in a population of American adults with MDD shows, once again, the indirect association between the concentrations of this element and other essential elements and depressive behavior [177]. The meta-analysis led by the same researcher suggests that there could be an inverse relationship between iron intake (Fe^2+^) and the risk of MDD [178]. Fe^2+^ levels are known to be closely associated with brain function [179]; for example, they are cofactors of tyrosine and tryptophan hydrolase, involved in the genesis of monoamines [180]. The functions of the striatum and the hippocampus are compromised by Fe^2+^ deficiency, affecting even the embryonic stage of the individual [181]. Magnesium (Mg^2+^) is involved in many physiological processes in the human body; of these, its calcium antagonist power has been related to mood by blocking NMDA receptors; in the same way, it is involved in the monoaminergic and glutamatergic systems and increases BDNF expression and reduces systemic inflammation, among other things [182]. 

Importantly, plenty of studies have been found to be related to specific micronutrient deficiencies at reproductive age, postpartum and later life; all of them are to be regarded as potential risk factors for different manifestations of depression. For this reason, it is of note to consider the different moments of life in women to manage specific requirements they may need with the aim to improve their prognosis in the case of diagnosed MDD, or to prevent the onset.

### 3.3. Specific Micronutrient Deficiencies Related to Menstruation, PMS and PMDD

Approximately 20–25% of women have moderate to severe PMS; and it is estimated that 5% of women have been diagnosed with its most severe form, PMDD [183]. It should be noted that prevalence increases in adolescence [184]. Lower levels of calcium and Mg^2+^, but not vitamin D, have been observed in women with PMS compared to controls [185]. Based on these facts, there seems to be an association between cyclical changes in women and calcium levels, thus affecting patients with PMS. Generally, serum calcium concentration is lower in this group, being inversely correlated with the incidence and symptoms of PMS [186]. However, despite the fact that there is the possibility of a relationship between vitamin D concentrations in women and premenstrual disorders, the benefits of its prescription are still not clear [187].

On the other hand, zinc seems to play a prominent role not only in mental but also in physical health, as has been shown in several placebo-controlled trials [188,189]. The review carried out by McCabe et al. shows the possible effectiveness of essential fatty acids in reducing premenstrual anxiety, having detected positive effects with the combination of vitamin B6 and Mg^2+^ [190]. To date, only one study has determined the relationship between iron and premenstrual syndrome; it is intuited that the mechanism that links them could be related to hormonal fluctuations, as happens in PPD. Having said that, the authors were able to affirm that high intake of non-heme iron is positively related to the reduction of premenstrual symptoms. In addition, the opposite occurred with high amounts of potassium, which produced an increased risk of PMS, possibly due to its aldosterone agonist power, which consequently increases fluid retention [191]. We cannot ignore the changes in appetite during the different phases of the menstrual cycle; estrogens and progestogens participate in this [192]. Due to the fact that patients with PMDD are more sensitive to hormonal variations, it has been concluded that there is an increased desire for sweet foods in the late luteal phase; this does not happen with foods with high salt content. It is proposed to focus on this fact to achieve new strategies that help in the clinical intervention of women with PMDD [193].

### 3.4. Specific Micronutrient Deficiencies Related to Perinatal and Postpartum Depression

There is a clear link between disruptions in both neurotransmitter and hormonal levels and fluctuations in micronutrients in women with PPD; therefore, it is important to know their influence and the antidepressant power of each one, with the aim to find alternative therapies with optimal effects. It is desired to obtain null adverse effects for lactation or ability to create addiction [194], which, a priori, will have a positive reception by patients in this group due to the harmless effect on their babies [195].

Several cohort studies suggest low levels of the circulating form of vitamin D, that is, 25-hydroxyvitamin D (25-[OH]-D), in patients with PPD [196,197]. The interdependence of vitamin D with calcium is widely known, and it is intuited that it plays an important role in the matter in question, since calcium stimulates signaling for the activation of gonadotropin, which in turn will lead to an increase in luteinizing and follicle-stimulating hormone which, as a consequence, increases the production of estradiol (E2). After childbirth, the demand for calcium increases and, with it, the demand for vitamin D. However, a study in which supplementation with vitamin D and calcium was combined in women with PPD versus placebo concluded that it cannot be stated that vitamin D decreases the severity of symptoms through increased E2 [198]. It should be noted that vitamin D could play an important role in improving depression in this group, but the lack of studies casts uncertainty on whether its effect is due to the relationship in the HPA axis, serotonin, hormonal and inflammatory levels or other mechanisms involved in the pathophysiology of PPD [199,200,201]. The same facts accompany perinatal depression [200,201].

Pregnancy is accompanied by increased blood cell volume and fetal and placental development, suggesting a sharp increase in Fe^2+^ demand [202]. A cross-sectional study with 142 pregnant women of more than 20 weeks, all of them with a history of mental disorders, such as anxiety or depressive episodes, concluded that there is a relationship between iron deficiency and perinatal depression [203]. The same thing happened in a study related to PPD, again evidencing this inverse relationship [204]. In contrast, the research carried out by Armony-Sivan et al. found no relationship between these two variables [205]. There is divergence in the results; however, there seems to be a significant relationship between low concentrations of hemoglobin or iron and the risk of suffering from depression after childbirth [206,207]. As in previous cases, it has been suggested that the decrease in ω-3 PUFAs levels is a factor that encourages the development of PPD. Women with an index greater than 5% of ω-3 during pregnancy have a lower risk of suffering from depression during the following year after birth [208]. However, a study of a cohort of Japanese women found no benefit of EPA and DHA supplementation in reducing risk [209]. Generally, prescription is not recommended as the main therapy, but it is considered a good complement [210]. NMDA receptor antagonist micronutrients such as Zn and Mg^2+^ have also been studied in this area, although it is true that the results have not been significant in improving symptoms in patients treated versus placebo [211], or a very slight positive effect has been seen [212]. However, in animal models, it seems that the combination of these microelements reduces the percentage of depressive symptoms after birth [213], which turns out to be encouraging data in order to continue focusing on the research on these micronutrients.

### 3.5. Specific Micronutrient Deficiencies Related to Peri- and Postmenopausal Depression

Perimenopause is considered a stage of high vulnerability for the development of depressive symptoms or behaviors, even in women without precedent [214]. The same thing happens in the post-menopausal phase [215]. 

Serum levels of some micronutrients such as vitamin D, iron, calcium and Mg^2+^ are lower in women with postmenopausal depression. Vitamin D deficiency is correlated with an increased incidence of changes in bone mass, commonly related to menopause [216]. In perimenopause, a negative association between vitamin D levels and depressive symptoms has also been found [217]. The deficiency is mainly due to low intake in the diet, little exposure to the sun due to the limitation of outdoor activities and reduced capacity for calcitriol synthesis [218]. Another trace element whose values are above the norm in postmenopausal women is copper, which, combined with the low concentration of Mg^2+^, seems to increase vulnerability to depression [219]. In contrast, the levels of vitamin B12 and folate are not altered in these depressive patients. ω-3 PUFAs have also been investigated in this field; it was determined that when the intake of these fats is higher, the prevalence is reduced, although the causes are unknown [220]. 

**Figure 3:**
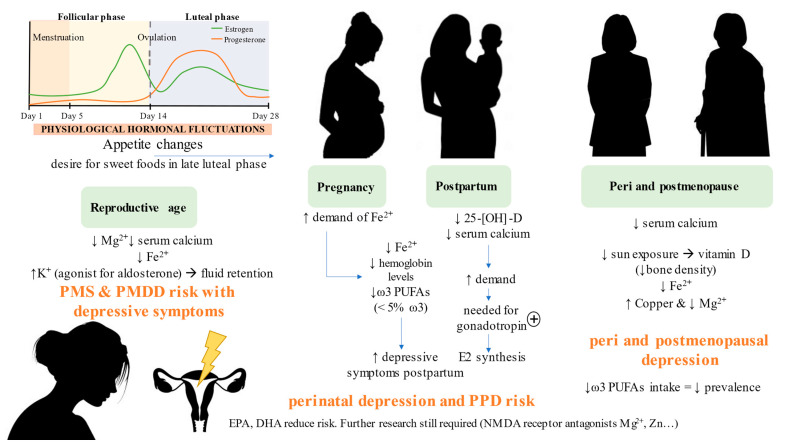
Nutritional deficiencies observed at different moments in a woman’s life that are associated with depressive symptoms. As summarized, there is in general a poor dietary context in women with depression, characterized by low fiber intake, high refined carbohydrates and sugar, unhealthy fats and low-quality protein intake, with detrimental effects on the brain. The improper dietary context is also related to several micronutritional deficiencies in women, with some particularities depending on the moment of their lives. MS: premenstrual syndrome; PMDD: premenstrual dysphoric disorder, PPD: postpartum depression; E2: estradiol.

All these common nutritional deficiencies observed in women with MDD at different ages are summarized in Figure 3.

## 4. Nutritional Intervention in Women with MDD

Addressing the nutritional status of patients with depression can be a profitable tool to aid in the possible pharmacological and psychological treatment of MDD, as well as prevention. Next, some of the most relevant findings will be detailed in relation to nutrients and bioactive components that have been gathered as evidence in clinical trials, in an attempt to translate the results by specifically focusing on women with depression. In this line, the concept of nutraceuticals stands out, which encompasses those food components that are essential and/or beneficial for the pathophysiology of the patient [221]. 

### 4.1. Fatty Acids

The human body does not synthesize fatty acids by itself, so it is necessary to incorporate them into the diet; the adequate amount of omega 3 should be 250 mg daily. The foods that most contribute this molecule are of both plant and animal origin; among them are chia seeds, soybeans, flax, nuts, vegetable oils, oily fish and shellfish [222]. There is strong evidence for the benefit of ω-3 PUFAs in depression [223,224]. The increase in EPA and DHA in cell walls correlates with the improvement of depressive symptoms and greater treatment effect [225]. Similarly, their antidepressant effect could be their anti-inflammatory and serotonin-modulating power, among others. Proinflammatory cytokines and BDNF mediate serotonin neurotransmission; cyclic AMP-responsive element-binding protein (CREB) has been found to be key to BDNF activation [226] which, as a consequence, translates into increased serotonin synthesis and reduced TNF-α, IL-6 and other proinflammatory cytokines. DHA and EPA modulate the levels of the neurotransmitter by this pathway [227]. Likewise, a study with ovariectomized rats investigated the synergy of estradiol and ω-3 PUFA, determining its antidepressant effects by the same route described previously. In addition, estradiol produced the regulation of glutamate, myo-inositol and cerebral glucose, which is related to less neuroinflammation and reduced neuronal death promoted by Glu [227,228]. The supplementation of ω-3 FA has been suggested in pregnant and lactating women; animal studies have shown a transfer of 25% of brain DHA to the fetus, which translates into a progressive depletion of the resource if the intake is not adequate; as a consequence, there would be a disruption of the release, reuptake and transmission of serotonin, as it is a main component of the terminal synaptic membranes. EPA correction, mainly in postmenopausal women, is recommended, among other things, for providing an improvement in psychotic symptoms [222]. 

### 4.2. Vitamins

#### 4.2.1. Vitamin D

The way in which we can obtain more of this fat-soluble molecule is by exposing the skin to ultraviolet radiation from the sun. To a lesser extent, it is found in foods such as milk, fatty fish or some vegetables [23].

Due to the production of active forms of vitamin D and the presence of VDR in intestinal cells, the close interaction of this microelement and the intestine could be intuited. On the one hand, several animal studies show the joint action of the intestinal microbiota and 25-[OH]-D in the modulation of inflammatory pathways, since it has been suggested that it is due to interaction with certain microbial metabolites, such as, for example, butyrate [229]. Some facts that prove this are the ability of both to repress the proliferation of inflammatory cytokines and regulate the signaling of nuclear factor ĸB (NFkB), the latter involved in the gene expression of inflammatory and immune responses [230]. Likewise, the importance of vitamin D in maintaining the integrity of the intestinal barrier has been observed. The intestinal mucous layer restricts the passage of bacteria through the epithelium; vitamin D can maintain adequate levels of antimicrobial peptides, for example, favoring the proliferation of Treg which, consequently, will inhibit the expression of Th1 immune cells and Th17, activating response due to bacterial penetration of endothelial cells [231]. On the other hand, germ-free mice presented hypovitaminosis and hypocalcemia, which were reversed with the inoculation of the intestinal microbiota, which suggests the direct effect of the microbiota on the metabolism of vitamin D [232]. 

In summary, vitamin D deficiency in patients with MDD could lead to increased permeability of the intestinal barrier, favoring endotoxemia and the consequent inflammation; likewise, the dysbiosis of microorganisms contributes to a decrease in vitamin D concentration, producing the attenuation of processes that contribute negatively to the pathophysiology of depression. In the case of women, the stages surrounding menopause and post-menopause are detrimental to the balance of bacterial species in the intestinal tract [233]. Supplementation with vitamin D3 would lead to a balance of microorganisms and a reduction in inflammation resulting from sudden estrogenic changes. The influence of this nutraceutical on quality of life, sleep hygiene and mood seem to be related to different pathways. Calcitriol mediates the formation of serotonin by activating the synthesis of tryptophan hydrolase 2 in different brain regions. In addition, its deficiency can affect the serotonergic system in another way; possibly melatonin and serotonin share common mechanisms; sun exposure and being able to influence mood through sleep quality may be important in MDD [234]. In relation to the above, the decrease in sleep quality, loss of energy and changes in eating habits in pregnant women or in postpartum periods are well known. The cohort study that was carried out in 890 women in the middle of their pregnancy used measurements of 25-[OH]-D in plasma, determining levels < 50 nmol/L to be insufficient; in addition, sleep patterns were monitored with the sleep quality index of Pittsburgh (PSQI), as well as the moment of the consumption of the highest percentage of calories (day or night). The results determined that insufficiency of 25-[OH]-D increases the risk of eating at night and the detriment of the quality of rest [235]. However, there is controversy about the relationship between vitamin D levels and the risk of PPD [236].

This nutraceutical not only seems to have an impact on the aforementioned; in research carried out in a population with a sample size of 897 adolescent girls, both without problems and with some condition associated with the menstrual cycle, they were given 50,000 IU/week of cholecalciferol during the nine weeks that the intervention lasted. Supplementation led to a drop in the prevalence of PMS and dysmenorrhea, from 14.9% to 4.8%, and 35.9% decreasing to 32.4%, respectively, as well as associated psychological and physical symptoms [237]. A clinical trial also observed the positive effect of vitamin D supplementation, this time combined with probiotics, on women with polycystic ovary syndrome [238]; this endocrine disorder appears to be linked to depression and anxiety [239]. 

#### 4.2.2. Vitamin B

Vitamin B deficiency seems to be a risk factor for the development of brain disorders such as cognitive disorders or dementia, due to its essential role in the folate and methionine cycles involved in nucleic acid methylation and homocysteine regulation. In addition, they are important cofactors in the biosynthesis of phospholipids and neurotransmitters [240]. Vitamin B9, also known as folic acid or folate (anionic form) is involved in the one-carbon cycle through the methionine cycle. Its active form, 5-methyltetrahydrofolate (5-MTHF), is crucial for the conversion of homocysteine to methionine through a reaction catalyzed by vitamin B12 and methionine synthase [241]. Likewise, methionine is the precursor for the production of SAMe, an important methyl donor for histones and DNA. Low levels of vitamin B stimulate the production of homocysteine [242]. These facts are closely linked with the menstrual cycle, assuming constant regeneration of endothelial tissue and follicular development; in this sense, a clinical study observed, by means of senescence markers p16, p21 and p53, the importance of folate as an antagonist of homocysteine in cell proliferation [243]. In accordance with the above, Michels et al. suggest that high homocysteine concentrations are related to altered hormonal changes and sporadic anovulation [244], abnormalities associated with polycystic ovary syndrome (PCOS) [245], assuming a risk factor for depressive symptoms and other psychiatric disorders [246]. Supplementation of B vitamins is suggested as a treatment to improve ovulation and fertility [244]. The prescription of folic acid during pregnancy is well known, among other things, because of its importance in the development of the fetus and the formation of the neural tube. Recent research correlates this with a lower risk of suffering from psychotic symptoms during pregnancy; in fact, in the animal model that was given doses of 1 and 5 mg/kg of folic acid, this nutrient was postulated as a potential contributor of cognitive alterations in the prenatal stage postpartum [247]; clinical trials agree with this evidence; thus its supplementation is recommended to reduce the risk of PPD [248]. This improvement appears to be linked to increased expression of pathways related to neuronal genesis, BDNF and synaptic transmission in the hippocampus [247]. 

#### 4.2.3. Minerals

Minerals play a key role in our development; therefore, they are considered essential molecules. They are divided into two: macromineral groups whose intake should be >100 mg/day and trace elements whose recommended dose ranges between 1 and 100 mg/day [249]. As previously mentioned, mineral levels are disturbed in patients with MDD; generally, there is a deficit of them, except for copper, which is in excess [250]. Magnesium is a macromineral highly implicated in the pathophysiology of neurological disorders [251]. In a clinical trial, differences were shown in the monitoring of participants for 12 weeks; for half of that time, they were supplemented with 248 mg/day of magnesium and the remaining time was for control. The findings were conclusive as to the effectiveness for the improvement of mild-moderate MDD without differences in age or sex; in addition, its low toxicity and rapid power of action were demonstrated [252]. In the research carried out on people with PCOS, 100 mg of magnesium, 4 mg zinc, 400 mg calcium plus 200 IU vitamin D were offered for 12 weeks compared to the placebo group; this had beneficial effects on hormonal profiles, biomarkers of inflammation and oxidative stress in women with PCOS [253]. The interest of this fact is the advantage it entails for the quality of life of the patient of childbearing age, thus being able to reduce the risk of the appearance of mental discomfort or depressive symptoms. 

For its part, iron is an important trace element that seems to be related to MDD. Although there is no solid evidence of the benefit of its supplementation in patients with MDD, the indications in animal tests justify future investigations. A randomized trial developed an experiment in which half of their patients were treated during the last 6 weeks of pregnancy with 50 mg of ferrous sulfate and the other half was the placebo group; the result was the improvement of PPD [254]. Additionally, the systematic review carried out by Lomagno et al. maintains that research in women would be of special importance due to the changes in levels such as those that can occur during menstruation, pregnancy or menopause. In this same study, the potential of zinc as a nutraceutical was also evaluated; in this case, the studies seem to point to the positive influence it has on mood regulation [255]. In the double-blind, placebo-controlled trial in young women, a multivitamin capsule and zinc 7 mg/day were combined for 10 weeks. The intake led to a reduction in scores related to anger, depression and discouragement along with an increase in serum zinc; these changes were not seen in the placebo group [256]. Accordingly, the general population with MDD could also obtain advantages, since the research review of patients treated with antidepressants plus supplementation versus placebo concluded that there was better response and recovery for those treated with this trace element [257]. Selenium is also a vital molecule for our body; the proper dose of it, along with other nutrients, can have a moderate impact on rectifying the development of TDM [258]. Furthermore, the possible protective effect on DPP has been suggested [259].

The inconsistency and scarcity of studies carried out on treatment with minerals justify the increase in research of this nature.

#### 4.2.4. S-Adenosyl Methionine

SAMe is the major known methyl donor in living organisms. It is a metabolite produced in the methionine cycle thanks to the enzyme methionine-adenosyl transferase (MAT2A), responsible for the transfer of adenosine to the sulfur of methionine, obtaining SAMe. This undergoes a series of transformations that leads to homocysteine; its remethylation mediated by 5-methyltetrahydrofolate will end up forming a new methionine molecule; this last step is connected to the folate cycle, vitamin B12 being necessary to complete the reaction in mammals [260]. The quality of donor of methyl groups makes SAMe a powerful mediator of epig6enetic mechanisms of both physiological functions in general and those related to the central nervous system due to its intervention in processes of transmethylation of DNA, histones, protein phosphatase 2A and catecholamine fractions. The alteration of one of the two cycles will contribute to the pathophysiology of psychiatric conditions, such as Alzheimer’s or MDD [261]. The synthesis of the cofactor tetrahydrobiopterin BH4 involved in the regulation of the production of monoaminergic neurotransmitters, together with the modulation of enzymes, transporters and monoaminergic receptors, is mediated by SAMe. It has been shown that patients with depressive disorders appear to have low levels of SAMe and MAT2A, which consequently compromises the previously described pathways [262], exacerbating the progress of the pathology. In addition, it is involved in inflammatory regulation and intestinal dysbiosis. A basic pillar of the anti-inflammatory action of SAMe against endotoxins is the downregulation of the expression of TNFα in immune cells (monocytes or macrophages) derived from the transcriptional induction promoted by lipopolysaccharide [263]. 

Mischoulon et al. demonstrated in their trial the improvement of cognitive impairment and depressive symptomatology using 800–1600 mg/day of SAMe for 6 weeks as an adjunct to antidepressant drugs [264]. It could even be recommended in patients with resistance to treatment. In addition, a plethora of clinical trials have studied its efficacy as monotherapy, showing positive effects against placebo and, in comparison, with other antidepressants [265]. However, sometimes it has not turned out to be as beneficial as expected [266]. There is gender-dependent divergence regarding the effectiveness of the nutraceutical, since the efficacy of the treatment led to a significant improvement in the severity of depressive symptoms measured with HAM-D in the sample of male subjects, while no differences were observed in women [267]. It would be convenient to investigate the mechanisms involved behind this difference in order to optimize the treatment of patients. The lack of studies in the female population with depression hinders the possible advantages that SAMe supplementation can provide in the specific alterations of this group.

In summary, the prescription of SAMe as a supplement in patients with MDD seems to be beneficial, mainly when it acts together with antidepressants; however, the particularities of each patient should be studied before doing so. The need to delve into the mechanisms of which SAMe is a part can be glimpsed for future research.

#### 4.2.5. Creatine and Amino Acids

Creatine (N-aminoiminomethyl-N-methyl glycine) is an endogenous nitrogenous molecule composed of amino acids. Its synthesis occurs in the liver, pancreas and kidney, but this supplies only half of the necessary amount, so its intake in the diet is essential, through foods such as red meat and fish, or by supplementation. It is known for its ergogenic benefits in athletes; however, recent research shows its interest in other areas [268]. Arginine, methionine and glycine are the amino acids necessary for its synthesis. The amidino group of arginine is transferred to the glycine molecule whose products will be ornithine and guanidinoacetate; then, SAMe donates a methyl group to guanidinoacetate to form creatine [269]. Creatine is mainly found in tissues with high energy demand due to its transformation into derivatives with great potential as rapid energy intermediates, such as phosphocreatine [268]. Compromise in the ability to metabolize creatine and alteration of serum levels in patients with MDD have been demonstrated [270], as well as the relationship of these with low intake from the diet [271]. Both preclinical and clinical studies demonstrate the antidepressant power of creatine due to its involvement in different pathways [270].

An animal model suggests sex-dependent differences in the effect of creatine supplementation, assuming an improvement in depressive behavior in females [272]. Consistent with this finding, the clinical study conducted on a group of women of different ages suggested that this protein improves the effects of antidepressant drugs, increases the connection of neural networks, which are attenuated in people with these conditions, and increases levels of N-acetyl aspartate in the prefrontal cortex, indicative of the improvement in neuronal integrity [273]. Likewise, dopaminergic and serotonergic neurotransmitters are increased with the presence of creatine detected in the cerebrospinal fluid [274]. Furthermore, modulation of sex hormones could affect creatine homeostasis. The difference in serum creatine kinase levels according to cycle phase and age has been observed; these increase in the luteal phase of the menstrual cycle synchronous with fluctuations in estrogen concentrations; however, they decrease during pregnancy and menopause. Creatine supplementation leads to improvements of various kinds, such as increased muscle tone and bone strength in menopause, lower risk of brain damage during pregnancy, as well as preservation of sleep patterns and cognition [274].

Depressed patients have reduced amino acid levels [275]; it is therefore logical to think that they are a viable nutraceutical for MDD. As mentioned in previous sections, tryptophan promotes the release of serotonin, and this is where its effectiveness in the treatment of MDD lies. A randomized crossover study supported these claims as to the benefits of tryptophan supplementation reversing pre-treatment anxious and depressive symptoms [276]. In addition, the balanced diet together with the consumption of tryptophan suggested the improvement of the general mood in patients without affective pathologies [277]. There also seems to be divergence in the effectiveness of the tryptophan supplement according to sex, since it was observed that the S/S′ genotype in women is indicative of greater protection from neurodegeneration, decreased cortisol and consequent mood repair. This effect was not seen in men [278]; however, more studies must be conducted before convincing recommendations can be given in this regard.

Along these lines, other amino acids such as tyrosine could also have antidepressant therapeutic properties, although the current literature is not conclusive [279].

#### 4.2.6. Bioactive Compounds

Various authors have debated the most approximate definition of a bioactive compound. Assessing the different points of view, Guaadaoui et al. [280] described it as “a compound that has the capacity and ability to interact with one or more components of living tissue, presenting a wide range of probable effects” We can extract them from plant, animal, microorganism or synthetic sources.

##### Phytoestrogens

They are polyphenols with functions similar to estrogens due to the similarity of their structures. Soy is the main source of this nutrient, although we can also find it in other legumes and vegetables. There are several benefits that phytoestrogens seem to have. Benefits have been observed in cancer, vascular diseases, osteoporosis and even in menopause. In the same way, it seems to have neuroprotective potential, related to its antioxidant power and its link with estrogen receptors [281].

Flavonoids, a type of phytoestrogens, positively intervene in neuroinflammatory processes, neurogenesis or GABAergic and monoaminergic transmission [282]. In humans, the consumption of orange juice rich in flavonoids (5.4 mg of flavonoids daily) for 8 weeks led to the modification of the intestinal microbiota, increasing species of the Lachnospiraceae family, correlated with the activation of BDNF signaling. Likewise, the evaluation of the Depression Scale of the Center for Epidemiological Studies noted an improvement in depressive symptoms [283]. Another clinical study, this time with women in the postnatal period, supports the facts stated above. Daily intervention with flavonoids over the course of two weeks showed significant differences between groups in the parameters of anxiety and perceived quality of life [284]. On the other hand, phytoestrogens have gained popularity among menopausal women, especially those who have certain doubts about hormone replacement therapy, due to the different advantages that their consumption entails. A meta-analysis determined that low doses of phytoestrogens (25 mg/d–100 mg/d) could relieve depressive symptoms [285]. However, the limitations of the studies make its use as monotherapy unfeasible; therefore, further research is necessary.

##### Caffeine

Caffeine is one of the most important bioactive components since it has neuroprotective effects. Although there are other compounds such as chlorogenic acid or caffeic acid, caffeine is the most widely used coffee nutraceutical [286]. Caffeine consumption has been linked to a lower risk of depression [287], in addition to the fact that some studies show that its consumption improves the effectiveness of antidepressant therapy [288], producing improvements in the treatment of motivational dysfunction and increasing dopamine levels as a result of the non-selective antagonist for adenosine A1/A2 receptors [289]. 

Similarly, in another study, positive results were obtained in those women with higher-than-average caffeine consumption (>261 mg), showing lower probability of developing dementia or other cognitive impairment disorders, unlike those who had lower than average consumption (<64 mg), indicating an inverse association between caffeine consumption and age-related cognitive decline [290]. These facts were supported in the longitudinal study by Lucas et al. [291]; in the study that was carried out with women divided by age, it caused an increase in blood pressure and a decrease in subjective feelings of mood, especially in those who were older. In addition, it was found that caffeine side effects were more likely in less physically active young women compared to physically active women [292]. Accordingly, its consumption is contraindicated in people suffering from PMS because it reduces the irritability and insomnia typical of this phase [293]. Finally, it should be noted that the dose of caffeine ingested affects some health factors of people with depression; high doses can generate mixed affective states, changes in the circadian rhythm, increased anxiety and/or dysregulation [294].

##### Anthocyanins

Anthocyanins are pigments from the group of flavonoids that we can find in red fruits, with blueberries being the largest natural source of this resource. In recent decades, their contribution to guarding against the risk of suffering from cardiovascular, neurological or diabetes diseases, among others, has been known [295]. The microorganisms of the intestine are involved in the complex degradation of this molecule at different levels. Likewise, its consumption will directly influence the colonizing species, since, according to the ample evidence provided in both in vitro, animal and human studies, an increase in beneficial flora such as *Lactobacillus* spp. and *Bifidobacterium* spp. inhibits the uncontrolled growth of harmful microorganisms. The ability to mold the microbial community and the precursor of short-chain fatty acids contributes to the possibility that anthocyanins also act as prebiotic agents [296]. In a study, the inflammatory improvement of the offspring of rats fed with different types of diets, one of them rich in anthocyanins, was verified. The results reflected the intervention of these flavonoids by decreasing the activation of the NFκB pathway, and expression of mRNA of TNF-α, IL-6 and proteins of the zona occludens (ZO-1), thus improving the general health of the digestive system [297].

On the other hand, their antioxidant power is widely known; in vitro studies and animal models highlight delphidin, cyanidin-3-glucoside and malvidin for their ability to bind oxygen radicals, be scavengers of superoxide anions and protect against oxidative stress [298].

Although it is true that the effect of this nutraceutical on depression has not been investigated, the clinical trial carried out on elderly adults with cognitive disorders, who ingested juice with a high content of anthocyanins (201 mg) for 8 weeks, obtained a reduction in serum TNF-α compared to the other groups, which may be indicative of cognitive improvement [299]. Another study supports this statement, since the magnetic resonance imaging tests reflected the increase in brain activity related to increased perfusion in regions responsible for this task [300]. Likewise, anthocyanin supplementation decreased the concentration of C-reactive protein in women with metabolic syndrome, along with improvements in cardiometabolic risk in the rest of the participants [301].

##### Resveratrol

Resveratrol (3,5,4′-trihydroxystilbene) is a polyphenol that can be found in red grapes, red wine and some nuts. Its appeal lies in its cytoprotective, antioxidant and anti-inflammatory power [302]. Recent studies have highlighted its beneficial effects on fatigue, sleep quality and anxious or depressive behaviors, such as anhedonia [303].

The effect of this flavone has been extensively studied in animal models. Resveratrol treatment of rats inoculated with high concentrations of corticosterone reversed the pathophysiological changes produced after 21 days of the trial, evidencing its interaction with the HPA axis [304]. Its anti-inflammatory power lies mainly in its ability to downregulate the expression of NF-kB, reduce the activation of microglia and reduce the proinflammatory response [303]. Likewise, the possibility of modulating processes of neuronal genesis was revealed, since it possibly regulates the activity of CREB, increasing the synthesis of BDNF [305]. The literature indicates that supplementation with this nutraceutical produces a decrease in monoamine oxidase (involved in the degradation of serotonin and norepinephrine) and, consequently, an increase in serotonin. In addition, the effect of the nutraceutical decreased in the absence of serotonin, proving its involvement in the monoamine system [306]. Tianyao et al. wanted to test the effect of resveratrol on behaviors similar to depression and anxiety due to lack of estrogen. Ovariectomized female mice were treated for 2 weeks with 20 mg/kg resveratrol. They concluded that estrogen deficiency inhibits the expression of Sirt (a key hippocampal protein for depression in rodents), an effect that was reversed by the action of resveratrol by inhibiting the NF-κB signaling pathway and the NLRP3 inflammasome in the hippocampus, which subsequently prevented inflammatory progress [307]. The estrogen deficiency in this trial simulates what could be the menopausal stage of women, so these encouraging findings put resveratrol in the spotlight as a possible antidepressant. Certainly, clinical trials of treatment with this polyphenol in women in this vital period have confirmed its benefit for health, reducing the pain related to the loss of bone mass, improving somatic symptoms, vaginal dryness, sleep and circulation disorders, in short, bringing about an improvement in overall quality of life [308,309].

Despite all the possible benefits found in animal models, clinical trials have not shown significant differences in the use of this nutraceutical [310]; however, the scarcity of human studies regarding this molecule cannot be ignored. Because of this, we suggest future research to optimize its effectiveness on health.

##### Cannabidiol

One of the bioactive compounds whose application is booming in recent times is cannabidiol (CBD), which is subtracted from the *Cannabis sativa* plant. This plant contains a large number of molecules known as phytocannabinoids; some of these, for example, tetrahydrocannabinol (THC), have psychotropic activity, producing physiological alterations after consumption. However, CBD does not have psychoactive properties and its toxicity is very low, so its use has been investigated for the treatment of different diseases such as epilepsy, chronic pain or Parkinson’s disease [311]. Among the mechanisms that link CBD and depression are the stimulation of neurogenesis and neuroplasticity in brain regions related to MDD [312]. Likewise, its participation in neurotransmission systems such as serotonergic, GABAergic and glutamatergic has the power to mitigate anxiety and decrease nausea [311]. Despite the facts, the lack of clinical evidence in patients with MDD or patients who have other particularities that could increase the risk of developing MDD in women (endometriosis or loss of quality of life in pregnancy) means that its use cannot be recommended [313,314,315]. It can be concluded that future research is necessary to discover the advantages that this nutraceutical provides in the particularities of the MDD pathology.

#### 4.2.7. Probiotics and Prebiotics

Probiotics are considered to be the set of live bacteria capable of providing health benefits; we find them in the form of supplements or in some fermented foods such as yogurt or kefir. By contrast, prebiotics are substrates that will act as nutrients for these microorganisms, causing a specific metabolism that results in a healthy ecosystem within the host. Currently, they are beginning to be focused on in order to use them as a key tool for the prevention and treatment of neuroimmune and neuroinflammatory disorders [316]. Due to this, the concept of “psychobiotics” arose, which corresponds to bacterial metabolites with regulatory activity of neurotrophic factors against the disruption of the gut-brain axis. Treatment with strains modified by genetic engineering, such as *Bifidobacterium breve* CCFM1025, recomposed the lost microbiota, increasing the production of short-chain FA, BDFN and serotonin [317]. In addition, the OXT/AVP complex can be regulated by intestinal microorganisms, with Lactobacillus reuteri being one of the main regulators known to date, favoring social behaviors [318]. Similarly, the efficacy of commercial formulas, composed of a combination of bacterial strains (*B. bifidum W23*, *B. lactis W51*, *L. brevis W63*, *L. casei W56*, *Lc. lactis W19*, among others), has been proven through striking data confirming the improvement of memory processes and sad mood in patients with mild or moderate MDD [319]. Women with PCOS were randomly divided into two groups; one of them was treated with a mixture of *Lactobacillus acidophilus*, *Lactobacillus reuteri*, *Lactobacillus fermentum* and *Bifidobacterium bifidum* plus 200 μg/day of selenium and the other was the placebo group. The results reflected the increase in glutathione and with it a greater antioxidant response, decreasing inflammatory markers such as C-reactive protein. In addition, the scores of the questionnaires were lower, a fact that shows improvement of mental health after treatment [320]. 

On the other hand, prebiotics do not seem to directly modify symptoms of anxiety or MDD according to the meta-analysis by Kazemi et al. [321]. However, the pro/prebiotic symbiosis can be considered key to the optimization of some strains. The use of these combinations has been transferred to the development of foods, from solids to beverages, with fermented foods being of great relevance. In this sense, it should be noted that food by itself does not provide benefits, since, for its determination, we must take into account the interaction between its components, together with its processing [319]. Due to the many limitations and lack of consistent facts in human studies, the benefits of consuming fermented foods cannot be assumed; however, preclinical trials point in the opposite direction [322], justifying the proposal that the investigation continues in this direction.

All these components are listed in Table 1, and their key aspects are also highlighted.

## 5. Conclusions

MDD is a rising condition affecting a large percentage of people worldwide, especially women, who present approximately 2-fold greater incidence than men. Despite the similarities, some critical differences in the pathophysiology and clinical presentation of MDD in women have been reported. Besides, there are specific subtypes of MDD in this group, including PPD or PMD. Besides, hormonal variations during the menstrual cycle can also be associated with the development of depressive symptoms, PMS and PMDD. Diet is a central element involved in the onset and progression of MDD, as many women affected by this condition appear to be malnourished, presenting inadequate macronutrient intake and deficits in several micronutrients. These alterations can be related to abnormal neurotransmission, negatively influencing sexual hormone levels and their actions. Furthermore, they may promote an inflammatory environment, with noteworthy gut dysbiosis, altering brain functioning and other pathophysiological mechanisms implicated in MDD. We encourage the promotion of integrative therapies for MDD patients from a sex-gender perspective with the aim to improve mental health in women since significant biological differences (menstrual cycle, pregnancy, menopause…) herein discussed are heavily associated with depressive symptoms. Nutritional programs constitute a profitable adjuvant to address MDD patients. Specific nutritional requirements have been observed at pregnancy, postpartum, menopause and even during hormonal fluctuations in the menstrual cycle. These deficiencies increase the risk of depression, with specific denominations according to the following life phases: depressive-like symptoms associated with PMS and PPDD; perinatal and PPD; peri and postmenopausal depression. Pharmacological treatment is sometimes inconceivable, especially during pregnancy and lactation. In this sense, addressing female MDD patients from a nutrition perspective would imply one step forward for precision medicine with a sex-gender perspective.

## Figures and Tables

**Table 1:** Nutritional components to consider in a nutrition intervention program for women with MDD.

Nutraceutical	Main Dietary Sources	Probable Antidepressant Effects	Clinical Evidence in MDD Patients	Specific Evidence in Women with MDD	References
ω-3 PUFAs	Nuts, seeds, oily fish and shellfish	EPA and DHA components of cell walls; neurotransmission and cell signaling; serotonin modulation via CREB; BDFN activation; anti-inflammatory; synergy with estradiol	Increased EPA and DHA in cell walls improve depressive symptoms and response to treatment; homeostasis ratio FA ω-6/ω-3 could be beneficial in patients with MDD	>5% FA ω-3 intake during pregnancy decreases the risk of PPD. Diets high in FA ω-3, mainly EPA, reduce prevalence of postmenopausal depression and improve depressive symptoms	[222,223,224,225,226,227,228]
Vitamin D	Oily fish, milk, some vegetables	Involved in neurological development; serotonin synthesis via tryptophan hydrolase 2; hippocampal integrity; balance in neuronal excitation and inhibition pathways; interaction with the intestinal microbiota, leading to joint action in the proinflammatory regulation and signaling of NFĸB; maintenance of antimicrobial peptides	-	Balance of the microbial ecosystem; reduction of inflammation produced by estrogenic fluctuations. <50 nmol/L of 25-[OH]-D in DPP alters sleep-wake patterns and timing of eating. 50,000 IU/week of cholecalciferol led to a drop in the incidence of PMS, dysmenorrhea and associated psychological and physical symptoms. Improves symptoms associated with PCOS	[23,229,230,231,232,233,234,235,236,237,238,239]
Vitamin B (B6, B9, B12)	Spices, nuts, liver, fish, meat, soybeans, vegetables, endogenous synthesis, supplementation	Involved in methylation processes; neurotransmitters and phospholipids synthesis; anti-inflammatory effect; homocysteine antagonist; SAMe precursor	-	Treatment for the improvement of alterations typical of PCOS. Folate supplementation during pregnancy decreases the risk of PPD	[240,241,242,243,244,245,246,247]
SAMe	Endogenous synthesis or supplementation	Power methylation processes; regulation of monoamine synthesis; anti-inflammatory effects; relation to the gut-brain axis	Effective adjuvant of antidepressants, also in patients with treatment resistance. Another trial determined the use of SAMe as a viable monotherapy	No improvements in severity measured with the Hamilton scale are observed in women. Scarce research makes it difficult to determine its possible benefits in this group	[260,261,262,263,264,265,266,267]
Magnesium	Whole grains, green leafy vegetables, nuts	NMDA antagonist; antioxidant; anti-inflammatory; involved in neurogenesis (BDNF)	248 mg/day of Mg improves mild-moderate MDD with rapid power of action and low toxicity	100 mg magnesium, 4 mg zinc, 400 mg calcium plus 200 IU vitamin D for 12 weeks had beneficial effects on hormonal profiles, biomarkers of inflammation, and oxidative stress in women with PCOS.	[251,252,253]
Iron	Legumes, seeds, seafood	Tyrosine and tryptophan cofactor; monoamine synthesis	-	50 mg of ferrous sulfate can improve PPD	[254,255]
Zinc	Meat, seafood, egg, some vegetables	NMDA antagonist; interaction with monoamines; neuroplasticity	Antidepressants together with zinc provides greater efficacy	7 mg/day of Zn + 1 multi-vitamin capsule for 7 weeks reduces anger, depression and discouragement.	[255,256,257]
Selenium	Nuts, red meat, grains, garlic	Antioxidant: anti-inflammatory	Diets with adequate amounts of Se and other micronutrients have a moderate impact on the inhibition of development of MDD	Possible protective effect of DPP	[258,259]
Tryptophan	Legumes, nuts, seeds	Serotonin synthesis	Reversal of anxious and depressive symptoms after treatment. Balanced diet plus tryptophan improves mood in healthy people	Sex-dependent differences, genotype S/S′ in women presents greater neuroprotection, decreased cortisol and consequent mood repair.	[276,277,278]
Creatine	Red meat, fish	Synthesis of energy intermediaries	Several clinical studies show the improvement of the pathology after supplementation	Sex-dependent differences: coadjuvant of antidepressant drugs, improvement of neuronal integrity and increased connection of neural networks. In another study, the increase in creatine was directly linked to a higher level of monoamines. Muscle and bone fortification, protection from brain damage in pregnancy and preservation of cognition and sleep hygiene	[268,269,270,271,272,273,274]
Phytoestrogens	Soy, legumes, vegetables	Antioxidant; anti-inflammatory; GABAergic and monoaminergic genesis and transmission; regulation of the gut microbiota	5.4 mg/day of flavonoids improves depressive symptoms and increases the Lachnospiraceae species, implicated in the activation of BDNF	Flavone supplementation in the postnatal period showed benefits in parameters of anxiety and quality of life. Doses of 25 mg/day–100 mg/day of phytoestrogens could alleviate depressive symptoms	[281,282,283,284,285]
Caffeine	Coffee, tea	Neuroprotection; dopaminergic regulation	Its consumption reduces the risk of depression and increases the effectiveness of pharmacological treatment. High doses could alter some factors related to health (circadian rhythms, anxiety, mixed affective states)	Differences in efficacy according to age: Doses > 261 mg of caffeine reduce the risk of developing dementia and cognitive disorders in later life. In young women, its benefit is not so decisive. Reduces irritability and insomnia in PMS.	[286,287,288,289,290,291,292,293,294]
Anthocyanins	Red fruits, red apple, cherry, black soybeans	Bidirectional relationship with the intestinal microbiota; possible prebiotic agent; anti-inflammatory; antioxidant	Increased beneficial flora (*Lactobacillus* spp. and *Bifidobacterium* spp.) 201 mg/day of anthocyanins produced the reduction of TNF-α, related to cognitive improvement	Decreased C-reactive protein in women with metabolic syndrome	[295,296,297,298,299,300,301]
Resveratrol	Red grape, red wine, nuts	Antioxidant; anti-inflammatory; cryoprotective; interaction with the HPA axis; neurogenesis; involved in the regulation of monoamines	Clinical trials demonstrate its power to improve fatigue, anhedonia or sleep quality. There is no specific evidence of improvement of MDD in humans	Increased quality of life, benefit in somatic and physical symptoms in menopausal women	[302,303,304,305,306,307,308,309]
CBD	Supplements	Implicated in neurogenesis and neuroplasticity; neurotransmission	-	-	[311,312,313,314,315]
Prebiotics and probiotics	Yogurt, kefir, tempeh, miso, legumes, fruits, vegetables	Benefits for the development of a healthy bacterial ecosystem; modulation of neurotrophic factors (BDNF); gut-brain axis regulation;	Supplementation with commercial formulas of probiotics improves mood and memory. Prebiotics by themselves do not intervene in the state of health, but they are key for the optimization of probiotics	In women with PCOS, the combination of bacteria of the genus *Lactobacillus* and *Bifidobacterium* +200 μg/day of selenium had antioxidant and anti-inflammatory power and resulted in higher scores on the mental health questionnaires.	[316,317,318,319,320,321,322]

## Data Availability

Not applicable.
